# Rethink of EGFR in Cancer With Its Kinase Independent Function on Board

**DOI:** 10.3389/fonc.2019.00800

**Published:** 2019-08-23

**Authors:** Rintu Thomas, Zhang Weihua

**Affiliations:** Department of Biology and Biochemistry, College of Natural Science and Mathematics, University of Houston, Houston, TX, United States

**Keywords:** EGFR, kinase independent function, mitophagy, cell survival, cancer

## Abstract

The epidermal growth factor receptor (EGFR) is one of most potent oncogenes that are commonly altered in cancers. As a receptor tyrosine kinase, EGFR's kinase activity has been serving as the primary target for developing cancer therapeutics, namely the EGFR inhibitors including small molecules targeting its ATP binding pocket and monoclonal antibodies targeting its ligand binding domains. EGFR inhibitors have produced impressive therapeutic benefits to responsive types of cancers. However, acquired and innate resistances have precluded current anti-EGFR agents from offering sustainable benefits to initially responsive cancers and benefits to EGFR-positive cancers that are innately resistant. Recent years have witnessed a realization that EGFR possesses kinase-independent (KID) pro-survival functions in cancer cells. This new knowledge has offered a different angle of understanding of EGFR in cancer and opened a new avenue of targeting EGFR for cancer therapy. There are already many excellent reviews on the role of EGFR with a focus on its kinase-dependent functions and mechanisms of resistance to EGFR targeted therapies. The present opinion aims to initiate a fresh discussion about the function of EGFR in cancer cells by laying out some unanswered questions pertaining to EGFR in cancer cells, by rethinking the unmet therapeutic challenges from a view of EGFR's KID function, and by proposing novel approaches to target the KID functions of EGFR for cancer treatment.

## Highlights

- EGFR possesses oncogenic pro-survival functions independent of its tyrosine kinase activity.
- Targeting EGFR's kinase independent functions may overcome cancer resistance to current EGFR inhibitors.

## Introduction

Structure-function based studies have firmly established the foundation of our knowledge about the canonical function of epidermal growth factor receptor (EGFR), a receptor tyrosine kinase that can dimerize, autocross-phosphorylate, and initiate a cascade of down-stream signals (1). Assuming that elevation of the default tyrosine kinase function of EGFR, owning to over-expression or kinase activating mutations, is all that cancer cells depend on in driving malignancy, the canonical tyrosine kinase function of EGFR has served as a beacon directing the design of EGFR targeted therapies for cancer. However, current potent EGFR inhibitors, small molecules of tyrosine kinase inhibitors (TKI) competing with ATP for kinase activation and monoclonal antibody inhibitors (mAb) preventing EGFR from being activated by its ligands, have exhibited limited efficacies and have been challenged by innate and acquired resistance in the clinic (2, 3). The majority of EGFR positive cancers do not respond to TKIs nor to mAbs, e.g., non-small lung cancers expressing wild-type EGFR, representing the innate resistance to TKIs. A fraction of EGFR positive cancers expressing EGFR with kinase activating mutation, non-small cell lung cancer (NSCLC) in particular, transiently respond to TKIs, however, these cancers develop acquired resistance to TKIs within about 1 year of therapy without exception, which exemplifies the acquired resistance (4–7). The exact mechanism underlying sensitivity to anti-EGFR mAbs remains undefined. Only a small fraction of EGFR positive cancers represented by advanced colorectal cancers expressing wild type KRAS respond to anti-EGFR mAbs although acquired resistance also commonly occurs (8, 9). The mechanism responsible for the innate resistance is largely unexplored.

The realization that EGFR possesses pro-survival functions independent of its kinase activity over the past decade has opened a new window for a better understanding the role of EGFR in cancer and offered a novel approach of targeting this powerful oncogene for cancer therapy.

## Alterations of EGFR in Cancer

EGFR is one of the most frequently altered oncogenes in solid cancers (1, 10). There are two types of pathological alterations of EGFR in cancers, one is kinase-activating mutation in EGFR and the other is over-expression of the EGFR protein. The kinase-activating mutations, which lead to increased tyrosine kinase activity of EGFR, can be primary or secondary to anti-EGFR therapies (11–13). Over-expression of EGFR protein can be associated with/without EGFR gene amplifications (14–21). Primary kinase-activating mutations in EGFR occur often in NSCLC and glioblastoma, but rarely in other types of cancers. In NSCLCs, EGFR is mutated in about 30–40% of East Asian patients and about 5–15% in non-East Asian patients (22, 23). In about 30% of glioblastomas, the 2–7 exons of EGFR are deleted which gives rise to an extracellular domain truncated EGFR named EGFRvIII whose tyrosine kinase is constantly active due to its ligand independent dimerization (24). As to secondary mutations contributing to the acquired resistance to anti-EGFR therapies, the T790M mutation accounts for 50% of resistance in NSCLC patients treated with first and second-generation TKIs (25–27). The C797S mutation is seen in T790M selective TKI treated NSCLC patients, however, its incidence remains unknown (28, 29). Mutations in the extracellular domain of EGFR were found in a few resistant colorectal cancer (CRC) patients after treatment with monoclonal antibody Cetuximab (30).

Unlike the EGFR kinase-activating mutations that occur mainly in NSCLC patients, wild-type EGFR protein is commonly over-expressed in many types of solid cancers and is often associated with negative prognosis (31–47), i.e., over-expression of wild-type EGFR is a more common phenomenon than EGFR mutations in solid cancers and promotes disease progression. Alterations of EGFR in 13 types of solid cancers and their responses to anti-EGFR agents are summarized in Table 1. It is worth noting that the majority of EGFR positive cancers do not respond to current anti-EGFR agents. Anti-EGFR therapies are mainly used for treating three types of cancers, which are NSCLC bearing kinase-activating mutations in EGFR for TKIs (4, 88, 89), about 10% of advanced metastatic colorectal cancers (CRCs) for anti-EGFR mAbs (90, 91), and locoregional advanced head and neck cancers (HNCs) for combination of mAbs with radiotherapy (92, 93).

**Table 1:** Alterations of EGFR in cancers and application of EGFR inhibitors.

**Cancer types**	**EGFR overexpression (%)**	**Activating mutations (%)**	**Application of TKIs**	**Application of mAbs**
**Lung**				
NSCLC	50–90 (34, 48, 49)	10–20 in not East Asian (50)	Yes^a^	No
		20–60 in East Asian (51)		
Prostate	40–100 (31)	Rare (52–54)	No	No
Breast	27–90 (55, 56)	Rare (57)	No	No
Colon and Rectum	80 (44, 58)	Rare (59)	No	Yes^c^
Head and Neck	90–95 (60)	Rare (61)	No	Yes^d^
Esophagogastric	27–44 (62, 63)	Rare (64)	No	No
Liver	68 (36, 37, 65)	Rare (66, 67)	No	No
Glioblastoma	40 (68, 69)	25 (24, 70, 71)	No	No
Cervix	54 (45)	Rare (72)	No	No
Ovary	30–70 (73, 74)	rare (75)	No	No
Bladder	70 (76)	Rare (77, 78)	No	No
Kidney	73–94 (79–81)	Rare (82)	No	No
Pancreas	65–95 (83–85)	Rare (86)	Yes^b^ (Marginal efficacy) (87)	No

a Marketed drugs: Gefitinib, Erlotinib, Icotinib, Afatinib, Decomitinib, Osimertinib, Olmutinib.

b Marketed drug: Erlotinib.

c Marketed drugs: Cetuximab, Panitumumab.

d Marketed drug: Cetuximab.

## The Mechanistic Basis for Current Anti-EGFR Cancer Therapies

Our understanding of EGFR started from the purification of the epidermal growth factor (EGF) (94), the default ligand of EGFR, the discovery of the intrinsic tyrosine kinase activity of EGFR (95), and the cloning of the EGFR gene (96). The canonical function of EGFR is initiated by ligand binding, which results in EGFR dimerization, cross-phosphorylation of its counterpart in the dimer at a few tyrosyl residues located at the carboxyl intracellular domain of EGFR, and subsequently these phosphorylated microdomains serve as docking sites for signal transductors to trigger downstream signaling cascades (1). This canonical mechanism of EGFR function has served as the authentic guidance for designing EGFR targeted therapeutics.

With regard to TKIs, there has been so called four generations of them. The first generation TKIs are represented by Gefitinib and Erlotinib, each of which reversibly competes with ATP to bind to EGFR (97). The second generation of TKI is represented by Afatinib that covalently binds to the ATP binding pocket to irreversibly inhibit EGFR's kinase activity regardless of EGFR mutations (98–100). The third generation of TKIs are represented by Osimertinib and Olmutinib that preferentially and covalently inhibit the T970M mutant of EGFR that is responsible for about 50% of acquired resistance to the earlier generation of TKIs (101–103). The fourth generation of TKIs, which preferentially inhibit the T790M/C797S EGFR mutant that leads to some resistance to the 3rd generation TKIs, are under early phases of preclinical development. The first 4th generation of TKI is represented by an allosteric inhibitor EAI045 that is effective in inhibiting the kinase activity of T790M/C797S only in combination with anti-EGFR mAb Cetuximab but not as a single agent (104), and its underlying mechanism is unknown. Updated molecular principals of design, action, and clinical impact of these TKIs have been comprehensively reviewed (105). *Regardless of the mutational selectivity of the TKIs, their effectiveness is determined by their capability of inhibiting the tyrosine kinase activity of a given form of EGFR*, i.e., *the tyrosine kinase activity of EGFR is the primary target*.

Currently, there are two FDA approved anti-EGFR mAbs for cancer therapies in the USA, Cetuximab and Panitumumab for metastatic colorectal cancer (9, 106, 107), and Cetuximab for locoregional head and neck cancer (92). The exact mechanisms underlying the therapeutic effects of the anti-EGFR mAbs remain to be defined, although the rationale for the design of these mAb is primarily rooted at blocking EGFR from being activated by its ligands (108–111). Proposed mechanisms mediating the therapeutic effects of anti-EGFR mAbs include inhibition of EGFR's kinase dependent downstream signals (108), down-regulation of membranous EGFR by induction of EGFR internalization and subsequent degradation in late endosomes (112), and induction of antibody-dependent cell-mediated cytotoxicity (113). However, neither the phosphorylation status nor the expression levels of EGFR in cancer tissues is predictive for efficacy of anti-EGFR mAbs (107, 114, 115). *The therapeutic effect of anti-EGFR Abs cannot be solely attributed to inhibition of EGFR's tyrosine kinase activity*.

### Persistent Challenges to Current Anti-EGFR Cancer Therapies

EGFR TKIs have clearly been clinically efficacious in responsive types of cancers (expressing kinase-activating mutations in EGFR), however, the benefits are often limited to improving the progression free survival (PSF) and quality of life rather than the overall survival (OS) (116–121). EGFR mAbs alone or in combination with chemotherapies have achieved an increase in unsustainable OS to <10% of metastatic colorectal cancer (122), and EGFR mAb in combination with radiotherapy has been shown to increase the 5 year OS rate by about 10% to regionally advanced head and neck cancers (123). Overall, meaningful clinical benefits offered by the current anti-EGFR agents are limited. Two major unmet challenges have stymied the efficacy of EGFR targeted cancer therapies.

The first challenge is the acquired resistance toward the anti-EGFR drugs, which has also been the research focus of EGFR targeted therapy. Expectedly, molecular adaptations at two levels, adaptive mutations in EGFR gene and adaptive gain-of-function of alternative survival and growth pathways play important roles in the development of acquired resistance to anti-EGFR drugs. As to TKI acquired resistance, reported resistant mechanisms include secondary amplification of and mutations in EGFR such as the T790M and C797S, gain-of-activities of alternative oncogenic pathways such as RAF/MEK/MAPK/ERK, PI3K/Akt, and MET regulated *signal* pathways, which has been extensively reviewed (4, 5, 88, 124, 125). Regarding anti-EGFR mAbs, mechanisms of acquired resistance are largely unclear, which is understandable given that the exact mechanism of action of these drugs has not been fully understood. Nevertheless, secondary mutations in the extracellular domain of EGFR, mutations in KRAS, NRAS and C-Met, loss of PTEN and activating mutations in PIK3CA, and gain-of-activity in the IGFR pathway have been associated with acquired resistances of some cases of colorectal cancer (8, 9, 126, 127).

The second challenge is the innate resistance to anti-EGFR drugs, which is much more prevalent than the acquired resistance. Although EGFR TKIs are potent in inhibiting the kinase activity of wild-type EGFR, cancers expressing wild-type EGFR, such as lung cancer (128–131), head and neck cancers (132), prostate cancer (133), and ovarian cancer (134), do not respond to TKIs regardless of the expression level of EGFR. In addition, NSCLCs with certain kinase activating exon 20 insertions are often insensitive to TKIs (135–138). There is about more than 80% of advanced colorectal cancers that do not respond to anti-EGFR mAbs (127). Many other types of EGFR positive cancers, such as prostate cancer (139, 140), and ovarian cancer (33) are innately resistant to anti-EGFR mAbs. One speculation has been that EGFR is simply unimportant for those cancers that are innately resistant to EGFR kinase inhibitor. This assumption has been negated by the observations of severe cell death upon down-regulating EGFR proteins in cancer cells of cancers innately resistant to EGFR kinase inhibitors, e.g., prostate cancer cells (141, 142), breast cancer, ovarian cancer cells, wild-type EGFR expressing lung cancer cells, wild-type EGFR expressing colon cancer cells (142–144), renal cancer (79), and glioma (145). In other words, *EGFR is indispensable for the survival of cancer cells that are innately resistant to EGFR kinase inhibitors*.

### Unanswered Questions Pertaining to EGFR's Kinase Dependent (KD) Role in Cancer Cells, and Their Impacts on Current Anti-EGFR Cancer Therapies

It has been more than a half century since the finding of EGF (94), the default physiological ligand of EGFR, from which the whole field of growth factors stemmed (95, 96). There is a large body of literature on EGFR biology which has firmly established the molecular mechanisms underlying its tyrosine kinase function and the canonical signal cascades governed by its kinase. However, when it comes to the utilization of these well-established theories to target EGFR for cancer therapy, the reality has raised some questions challenging the comprehensiveness of our knowledge on EGFR in cancer.

**Question 1**: Given the fact that increased protein expression level of EGFR correlates with cancer progression and over-expression of wild-type EGFR is tumorigenic, *why do wild-type EGFR expressing cancers not respond to TKIs*?

Our understanding of EGFR began from studying the function of wild-type EGFR using cancer cells (146, 147) and non-cancerous cells (148–151). Regardless of the cell types being used, TKIs have exhibited potent *in vitro* and *in vivo* effects on inhibiting the tyrosine kinase activity of wild-type EGFR. Over-expression of wild-type EGFR is tumorigenic in several types of cells (152–155), validating that wild-type EGFR is oncogenic. On one hand, protein levels of EGFR, but not its phosphorylation status, is strongly associated with disease progression and poor prognosis of many types of cancers that rarely express mutated EGFR (31, 32, 34, 38, 55, 76, 80, 156–160). Examples of cancers that exhibit increased EGFR expression along with disease progress and do not respond to TKIs include prostate cancer (133), ovarian cancer (157), pancreatic cancer (161), colorectal cancer (162), head and neck cancer (40), cervical cancer (163), and lung cancers expressing wild-type EGFR (128, 152, 164). On the other hand, EGFR mutations but not protein expression levels are associated with responsiveness to EGFR TKIs (165). *There is no doubt that wild-type EGFR protein promotes cancer progression, but why do cancers expressing wild-type EGFR not respond to EGFR TKIs?*

Explanation to this puzzling phenomenon has been that the wild-type EGFR expressing/overexpressing cancers are not addicted to EGFR function for growth/survival, however, this assertion is challenged by observations that TKIs are potent in inhibiting the growth of wild-type EGFR expressing cells (166–171) and by studies showing wild-type EGFR expressing cells cannot survive after EGFR knockdown by siRNA (142, 145, 172–174). These observations suggest that the EGFR wild-type cancer cells may be not addicted to EGFR's kinase activity but rely on the existence EGFR for survival, i.e., the survival of cancer cells is sustained by EGFR without involving its kinase activity.

**Question 2**: *Why does the phosphorylation status of EGFR not correlate with cancer progression nor with responsiveness to anti-EGFR drugs?*

There is no doubt that activation of the tyrosine kinase activity of EGFR, regardless of its mutational status, promotes cell proliferation and tumor growth of experimental models, which has served as the scientific basis supporting the targeting of the kinase activity of EGFR for cancer therapies (175). However, on one hand the level of total EGFR protein expression is closely associated with poor prognosis of many types of cancers including those cancers resistant to anti-EGFR agents (31, 32, 34, 38, 39, 80, 156–159) and gene copy number of EGFR is currently one of the most reliable predictors for sensitivity to anti-EGFR therapeutics; on the other hand, the level of phosphorylated EGFR is not a reliable predictor of NSCLC's sensitivity to TKIs and mutational status of EGFR is (89, 176, 177)—why?

The lack of association between pEGFR levels and disease status has been hypothetically attributed to a sum of technical inconsistences among studies, such as technical variations in performing immunohistochemistry, qualities of anti-pEGFR antibodies, procedures of cancer tissue preservation in the clinic, and patient sample size employed for analysis. These possibilities portray a virtually impossible mission to having these issues resolved. However, it does not stop the proposition of an untested concept that if the kinase activity of EGFR is indeed not critically involved in progression of cancers expressing wild-type EGFR but the total level of EGFR protein is, shouldn't we start considering a possibility that EGFR may own powerful oncogenic functions independent of its tyrosine kinase activity? This possibility is supported by a recent study that loss-of-function mutations of all the phosphorylatable tyrosyl residues of the C-terminal domain of a kinase-activating EGFR mutant retains its oncogenic function (178), i.e., the kinase dependent down-stream signaling of EGFR is not required for its oncogenic function.

**Question 3**: *Why do the TKI responsive cancers not overlap with the anti-EGFR mAbs responsive cancers?*

Both TKIs and anti-EGFR mAbs are potent in inhibiting the tyrosine kinase activity of EGFR in cancer cells, however, oddly the responsive cancer types of these two kinds of anti-EGFR reagents do not overlap at all. TKIs are approved for NSCLC especially for cancers expressing mutated EGFR (179, 180), whereas anti-EGFR mAbs are approved for KRAS wild-type colorectal cancer and local regional head and neck cancers (40, 106). Currently, there is no positive biomarker available for selection of cancer types that are favorable to anti-EGFR mAbs, although KRAS mutations are a negative predictor for anti-EGFR mAbs in treating colorectal cancer (181). An obvious question raised by this discrepancy between suitable cancer types of TKIs vs. mAbs is: Is inhibition of the kinase activity of EGFR primarily accountable for the efficacy of anti-EGFR mAbs?

**Question 4**: *Is the tyrosine kinase activity of EGFR the shared primary driver of EGFR's pro-growth and pro-survival functions?*

It has been a conventional statement that EGFR as a receptor tyrosine kinase plays important roles in promoting cell growth and survival without differentiating its weight on growth vs. survival; what is even more equivocal is that the EGFR regulated cell growth and survival has never been mechanistically differentiated. Cell growth and cell survival are totally different biological events, the former refers to increase in numbers or in size of individual entity whereas the latter refers to the ability to cope with stresses in order to stay alive. Growth depends on survival, however, survival is independent of growth. Cancer is a disease driven by abnormal cell growth and cell survival, thus treatment strategies ought to be differentially directed toward growth and survival.

Accumulated data over the past two decades strongly suggest that the tyrosine kinase activity of EGFR is predominantly involved in promoting cell proliferation (175) compared to cell survival. Consistently, EGFR TKIs and mAbs have constantly exhibited anti-proliferative effects under physiologically relevant conditions (182–185), which are often accompanied by surrogate makers of cell survival but not direct evidence of cell death of *in vitro* cultured cells (47, 166, 171, 186–196). Given the current understanding that the apoptosis process is reversible even at stages of the activation of caspases (197) and that therapeutic stresses can cause secretion of DNA fragment containing exosomes by cancer cells (198, 199), which can interfere the interpretation of the increase of sub-G0 cells (used to represent apoptotic cells using flow cytometry) caused by TKI treatments. Furthermore, TKIs or mAbs do not cause DNA fragmentation in many types of EGFR-positive cancer cells while their growth inhibition effects are obvious (166, 167, 169, 200–202). Regarding the impact of TKIs on cell survival, recent studies have revealed that TKIs are potent in inducing cytoprotective autophagy that in turn promotes cell survival (203–207).

It is critical to differentiate EGFR's pro-growth from its pro-survival functions, because if the kinase activity of EGFR is not pivotal for sustaining cancer cell survival, it becomes explainable that the current anti-EGFR reagents aiming to block the kinase activity of EGFR are unable to significantly induce death of cancer cells but are good at transiently inhibiting growth of cancer cells before cells develop alternative pro-growth signal pathways resulting in resistance. Re-growth associated gain of kinase activity mutations in EGFR (such as T709M and C797S) strongly suggests that the kinase activity of EGFR is important for cell growth. The dependence on EGFR for survival and the impact of its kinase activity on cell proliferation raises another important question: Are the pro-growth and pro-survival functions of EGFR divergent at its tyrosine kinase activity?

### Current Standing of Our Knowledge of EGFR Biology and EGFR Targeted Cancer Therapy

Our current understanding of EGFR's canonical function and status of its tyrosine kinase targeted cancer therapy can be summarized as follows:

Validated canonical functions and mechanisms of EGFR action:

1. The tyrosine kinase activity of EGFR and mechanisms connecting with its down-stream signal cascades
2. The growth promoting role of the tyrosine kinase activity of EGFR
3. The oncogenic capacity of EGFR
4. The positive association of EGFR expression with progression of certain cancers
5. The dependence of kinase-activating mutations in EGFR for therapeutic effect of TKIs
6. The dependence of kinase-activating mutations in EGFR for a portion of acquired TKI resistance
7. The fact of unavoidable resistance to current anti-EGFR therapeutics
8. The clinical benefit of increased progression free survival but not overall survival for patients suitable for treatment of TKIs.

Overarching challenges:

1. Why do wild-type EGFR expressing/overexpressing cancers not response to EGFR TKIs?
2. What is the exact mechanism underlying anti-EGFR mAbs' therapeutic effect?
3. What is the exact mechanism underlying EGFR's pro-survival function in cancer cells?

### Realization of the Existence of Kinase Independent Pro-survival Function of EGFR in Cancer Cells

As discussed above, when it comes to the question pertaining to EGFR's pro-survival function, the kinase activity of EGFR does not offer a full accountability. The past 10 years have witnessed a growing body of evidence indicating that EGFR possesses pro-survival functions that are independent of its tyrosine kinase activity in cancer cells.

In cancer cells, by comparison of the effects of an EGFR TKI and EGFR siRNA, it was found that EGFR maintained survival of prostate cancer cells independent of its kinase activity, i.e., TKI inhibited cell proliferation without effecting on cell survival whereas loss-of-EGFR expression induced by siRNA knockdown led to severe autophagic cell death that could be rescued by a kinase-dead EGFR (142). Furthermore, this study found that the sodium/glucose co-transporter 1 (SGLT1) played a critical role in mediating the KID pro-survival function of EGFR by maintaining active glucose uptake of cancer cells (142).

**Figure 1:**
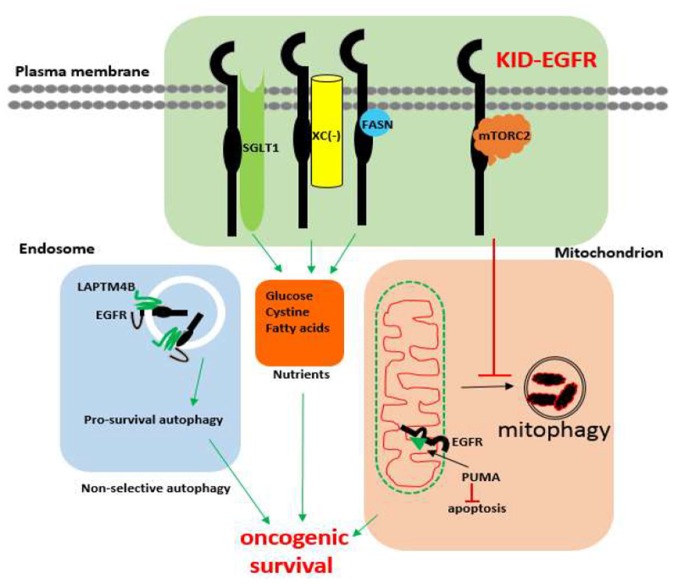
Known kinase-independent functions of EGFR in cancer cells. Currently known kinase-independent (KID) functions of EGFR locate at three functional domains of cancer cell. One is in the plasma membrane where EGFR interacts with SGLT1, Xc^−^, fatty acid synthase (FASN), and the mTORC2 complex to support transportation of glucose, cystine, *de novo* fatty acid synthase, and repressing mitophagy, respectively. The second function domain is the endosomal autophagy machinery where kinase inactive EGFR promotes pro-survival autophagy. The third domain is the mitochondrial domain where kinase inactive EGFR interacts with PUMA to inhibit apoptosis. KID-EGFR is oncogenic and pro-survival.

The existence of KID pro-survival function of EGFR has also been revealed in different types of cancer cells involving several cellular functional domains that include the plasma membrane, the autophagic machinery, and the mitochondrion. Within the plasma membrane, kinase independently, EGFR interacts with SGLT1 to maintain active glucose uptake (142), interacts with the system ${\text{x}}_{\text{c}}^{-}$ antiporter to maintain cystine import (145), interacts with fatty acid synthase to maintain *de novo* fatty acids synthesis (208), and interacts with the mTORC2 complex to suppress Akt (143). Within the autophagy domain, inhibition of the kinase activity of EGFR promotes pro-survival autophagy (205, 207, 209) and endosomal kinase inactive EGFR interacts with LAPTM4B to promote pro-survival autophagy under nutrient starvation stress (210). As for mitochondrion, kinase independently, EGFR inhibits mitophagy via repressing intracellular activation of Akt (143), and EGFR interacts with PUMA to inhibit apoptosis (211). An update on the kinase-independent functions of EGFR in cancer cells is summarized in Figure 1.

In non-cancerous cells, one of two kinase-impaired EGFR mutants was found to be able to oppose IL3-removal induced apoptosis of an EGFR negative noncancerous hematopoietic 32D by undefined mechanisms (212), and knockout of EGFR in mice is lethal (213) but mice with a loss-of-kinase mutation in EGFR are viable with only mild defects in the eyes and skin (214). The discrepant phenotypes of EGFR knockout mice and mice bearing loss-of-kinase mutant EGFR argues that EGFR also exhibits KID functions in non-cancerous cells, however, this is beyond the scope of this review. The existence of KID pro-survival function of EGFR is undeniable.

### Implication of KID Functions of EGFR in Advancing Our Understanding the Role of EGFR in Cancer

While much more research effort is needed to fully unveil the KID pro-survival function of EGFR in cancer cells, the discovery and realization of EGFR's KID pro-survival function bears a profound implication on overcoming the aforementioned long-lasting challenges of EGFR targeted cancer therapies.

**Figure 2:**
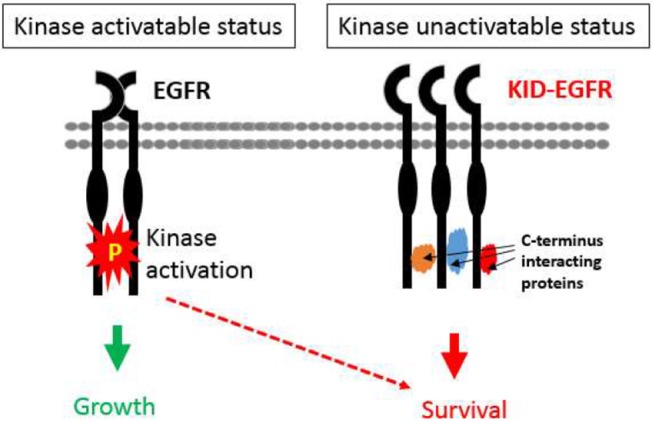
A model of two functional statuses of EGFR in cancer cells. The kinase activatable EGFRs are mainly involved in promoting cell growth, and the kinase unactivatable EGFRs, which are blocked from autocross-phosphorylation by interacting proteins, are mainly in charge of promoting oncogenic cell survival.

First of all, it offers an alternative interpretation to the clinical failures of EGFR kinase inhibitors. Regarding cancers expressing/overexpressing wild-type EGFR, such as head and neck, prostate, and ovarian cancer, which are innately resistant to EGFR TKIs, a new interpretation is that these types of cancers are more dependent on EGFR's KID function for survival rather than on its kinase activity for growth. This possibility is supported by the fact that, without TKI treatment, the phosphorylation status of EGFR does not correlate with disease progression nor with prognosis of many cancers but the total EGFR protein level does (31, 32, 34, 38, 39, 80, 156–159) and further that the innate TKI resistant cancer cells cannot survive without EGFR (141–143, 215), and that disconnecting the EGFR's kinase activity from its downstream kinase cascades does not affect EGFR's oncogenic function (178). Regarding the acquired TKI resistance, an alternative interpretation is that TKI treatment shifts EGFR's kinase dependent function toward its KID pro-survival function that offers cancer cells addicted to EGFR's kinase activity for growth an adaptive window to develop alternative proliferative mechanisms circumventing the EGFR kinase dominated pathway under the constant exposure to TKIs. This possibility is supported by the observation showing that in cancer cells EGFR exists in two types of status, a kinase activatable one and a kinase unactivatable one (216). The kinase activatable EGFR refers to the EGFRs that behave according to the canonical mechanisms, whereas the kinase unactivatable EGFR refers to the EGFRs physically interacting with other proteins at its C-terminal kinase domain, such as the EGFRs interacting with SGLT1 (216), thus cannot be autophosphorylated. Supportively, it has been recently reported that autophosphorylation of the C-terminal domain of EGFR is not required for EGFR's oncogenic activity (178). The shift toward KID function of EGFR by TKI in TKI sensitive cancer cells is also supported by the observations that TKIs shift EGFR from non-lipid raft regions to lipid rafts (217) where many cell survival dependent proteins, such as mTORC2, Na+/K+ ATPase, fatty acid synthase reside (218). Additionally, TKIs, especially the first generation of TKIs (Gefitinib and Erlotinib), are capable of causing dimerization of EGFR without significantly altering the level of EGFR protein (219–221) in a manner that is dependent on EGFR palmitoylation and independent of EGFR's kinase activity (222), which implicates that the TKI induced kinase inactivated EGFR dimer may gain new functions by recruiting novel interacting proteins. Further supports for the hypothesis that the kinase activity of EGFR is more critically involved in the proliferation than in the survival of cancer cells are offered by two studies: one is a study using rat models showing that inhibition of EGFR's kinase activity by TKI was able to inhibit growth but not the incidence of chemical or hormonal induced liver cancer (223), and consistently another study shows that the phosphorylation of the C-terminal tail (a hub domain that connects the kinase function of EGFR with its down-stream kinase dependent signaling cascades) is not required for the oncogenic function of EGFR mutant derived from lung cancer (178). Thus, we propose a new model of EGFR function: EGFR exists in two types of functional nodes, a kinase dependent functional node (the canonical functional node) that predominantly oversees cell proliferation and a kinase independent functional node that predominantly oversees cell survival, which is depicted by Figure 2.

The KID pro-survival function of EGFR also explains the worse prognosis of patients treated with a combination of EGFR TKI and chemotherapeutics than those treated with chemotherapeutics alone (164, 224–227). One explanation to it is that, in these scenario, the KID oncogenic function of EGFR might be enhanced by TKIs and thus survivability of cancer cells, which hampers the cytotoxic effect of the chemotherapeutics.

Secondly, the KID function of EGFR offers an alternative interpretation to experimental observations that cannot be fully explained by the kinase function of EGFR. For example, the contrasting phenotypes between EGFR knockout mice (213) and loss-of-kinase EGFR mutant mice (214), in the former model where the EGFR gene was systemically knocked out, the homozygous EGFR^−/−^ mice die within a week after birth due to failures of multiple organs especially the lung and the heart, whereas the Waved-2 mice who lose more than 99% of EGFR's tyrosine kinase activity, survive and develop well with only a minor defect in the hair follicles that give rise curved hairs. The discrepancies between these two animal models of EGFR mutant argue that the tyrosine kinase activity is not the sole physiological function of EGFR and the KID function of EGFR is critical for the survival, although a proof-of-concept definitive experiment of rescuing the EGFR knockout mice with a kinase dead form EGFR needs to be performed.

Thirdly, the KID function of EGFR offers a partial explanation for the unique therapeutic effect of the anti-EGFR mAbs. Neither the phosphorylation status nor that for the total EGFR expression are predictive of responses to anti-EGFR mAbs (107, 115, 177), suggesting that repression of the kinase activity of EGFR by these mAbs might not be the primary mechanism underlying the therapeutic effect of anti-EGFR mAbs. Unlike the TKIs that can induce EGFR dimerization without activation (219, 220, 228) and are only effective in cancers bearing kinase-activating mutations in EGFR, anti-EGFR mAbs are capable of reducing EGFR proteins by shifting the ligand induced EGFR endocytosis toward the non-recyclable stage, the late-endosomal stage where EGFR is to be degraded rather than being recycled back to the plasma membrane as most of the early-endosome localized EGFR are programed to do (9). Many studies have proposed that the mAb binding induced EGFR endocytosis and subsequent degradation is a key mechanism as compared to antigen dependent cellular cytotoxicity (ADCC) by which anti-EGFR mAbs execute their therapeutic effect. This is supported by the observation that both Cetuximab and Panitumumab are capable of reducing EGFR protein levels, however, unlike Cetuximab (229–231), Panitumumab, as an IgG2 is less capable of inducing ADCC (231). The EGFR endocytosis induced by anti-EGFR mAbs is not free of EGFR recycling, although the balance between degradation and recycling is tilted toward degradation as compared to the EGFR ligand binding induced endocytosis (232). A better understanding of mechanistic differences between ligand induced EGFR endocytosis and that induced by anti-EGFR mAb may lead to discovery of novel actionable targets to enhance the effect of mAb induced reduction of EGFR protein and the therapeutic efficacy of anti-EGFR mAbs.

While the specific mechanisms underlying the KID pro-survival function of EGFR remains to be fully revealed, existing evidence is sufficient in concluding that the pro-survival function of EGFR is regulated by mechanisms that are largely independent of EGFR's kinase function. It is proposed that targeting the KID pro-survival function of EGFR by reducing its protein levels or interrupting the mechanisms mediating its KID pro-survival function may lead to novel and more effective approaches of targeting EGFR for cancer therapy. In this regard, a proof-of-concept synthetic peptide that can cause degradation of EGFR has been shown to be effective in treating orthotopic ovarian cancers in mice by inducing mitophagic cell death of cancer cells (143).

### Perspective on EGFR Targeted Cancer Therapies

EGFR is the most commonly expressed/overexpressed membranous oncogenic protein in cancer. The majority of EGFR overexpressing cancer patients are yet to benefit from current anti-EGFR therapeutics. Targeting the kinase activity of EGFR is preordained to acquired and innate resistance. Given its frequent expression in cancers, its powerful oncogenic function, and easy accessibility for targeting, EGFR remains an ideal therapeutic target for cancers. A growing body of evidence has revealed that hijacking kinases for non-kinase usages by cells is a common phenomenon (233, 234). For cancers, besides EGFR, it has been found that, kinase independently, AKT promotes cancer cell survival (235), AURKA (Aurora kinase A) enhances stemness of breast cancer cells (236), cyclin-dependent kinase 6 promotes tumorigenesis of lymphoma (237), cyclin-dependent kinase 19 promotes cell proliferation of osteosarcoma cells (238), cyclin E promotes proliferation of liver cancer cells (239), EphA2 (ephrin type-A receptor 2) promotes invasion and metastasis of prostate cancer (240), ERKs promote cell cycle entry of retinoblastoma cells (241), PAK4 (P21-activated kinase 4) promotes adhesion and migration of breast cancer cells (242), and RIPK1 (receptor-interacting protein kinase 1) promotes liver carcinogenesis (243). The time is now to step out the box of the tyrosine kinase function of EGFR and explore new ways of targeting EGFR.

**Figure 3:**
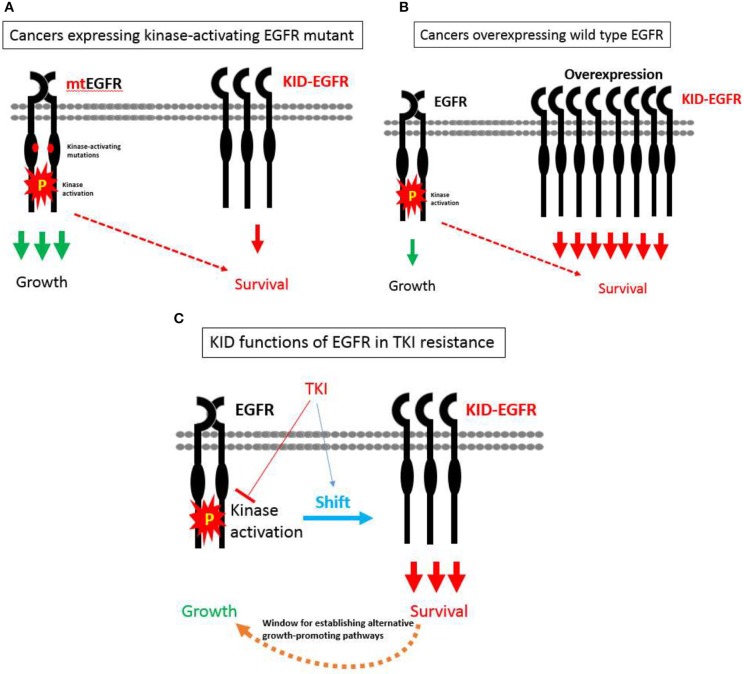
A hypothesis pertaining to EGFR's divergent roles in regulating growth vs. survival of cancer cells in relevant to TKI resistance. **(A)** In cancer cells expressing kinase-activating mutations, the role of EGFR is shifted toward its kinase-dependent functions, which sensitizes these cancers cells to TKI. **(B)** In cancer cells over-expressing wild type EGFR, the role of EGFR is shifted toward its kinase-independent functions, which promotes the progression of cancers rather desensitizes these cancers to TKI. **(C)** At situation of TKI treatment, the role of EGFR is also tilted toward its kinase-independent functions that allows cancer cells to survive and develop alternative growth-promoting mechanisms to counteract with TKI's inhibitory effect.

With the KID functions of EGFR on board, a hypothesis pertaining to EGFR's divergent roles in regulating growth vs. survival of cancer cells in relevant to TKI resistance is proposed as the following (Figure 3): EGFR exists in two types of status, one is kinase activatable and the other is kinase unactivatable (functions as a scaffold protein), the former is mainly in charge of cell growth, the latter is mainly in charge of survival. In cancer cells expressing kinase-activating mutations, the role of EGFR is shifted toward its kinase-dependent functions; while in cancer cells over-expressing wild type EGFR, the role of EGFR is shifted toward its kinase-independent functions; at situation of TKI treatment, the role of EGFR is also tilted toward its kinase-independent functions that allows cancer cells to survive and develop alternative growth-promoting mechanisms to counteract with TKI's inhibitory effect.

The evidence of KID function of EGFR is somewhat scattering however undeniable, and more researches on the KID functions of EGFR are warranted. Targeting EGFR's KID functions by either decreasing EGFR protein levels or interfering with the mechanisms underlying EGFR's KID functions forecast significant promise. Currently proposed approaches may include disrupting the protein-protein interacting complex of KID EGFR, down-regulating EGFR protein using synthetic molecules (143, 222), siRNA or protein targeting chimeras (PROTAC) technologies (244), and manipulating signal pathways controlled by KID EGFR such as simultaneously activating mTORC2 and inhibiting mTORC1 (143).

## Author Contributions

ZW formulated the concept. ZW and RT co-wrote the manuscript.

### Conflict of Interest Statement

ZW is a co-founder and a shareholder of Metabocentric Biotechnologies Inc., a spinoff startup company of the University of Houston, which focuses on developing metabolic therapeutics for cancer. The remaining author declares that the research was conducted in the absence of any commercial or financial relationships that could be construed as a potential conflict of interest.
